# Pit Latrine Fecal Sludge Resistance Using a Dynamic Cone Penetrometer in Low Income Areas in Mzuzu City, Malawi

**DOI:** 10.3390/ijerph14020087

**Published:** 2017-02-03

**Authors:** Charles F. C. Chirwa, Ralph P. Hall, Leigh-Anne H. Krometis, Eric A. Vance, Adam Edwards, Ting Guan, Rochelle H. Holm

**Affiliations:** 1 Centre of Excellence in Water and Sanitation, Mzuzu University, Private Bag 201, Mzuzu 2, Malawi; fcharlesc@yahoo.com; 2 School of Public and International Affairs, Virginia Polytechnic Institute and State University, Blacksburg, VA 24061, USA; rphall@vt.edu (R.P.H.); lehenry@vt.edu (L.-A.H.K.); 3 Department of Applied Mathematics, University of Colorado Boulder, Boulder, CO 80309, USA; Eric.Vance@colorado.edu; 4 Department of Statistics, Virginia Polytechnic Institute and State University, Blacksburg, VA 24061, USA; adaedwar@vt.edu (A.E.); ting@vt.edu (T.G.)

**Keywords:** developing countries, fecal sludge management, peri-urban, sanitation

## Abstract

Pit latrines can provide improved household sanitation, but without effective and inexpensive emptying options, they are often abandoned once full and may pose a public health threat. Emptying techniques can be difficult, as the sludge contents of each pit latrine are different. The design of effective emptying techniques (e.g., pumps) is limited by a lack of data characterizing typical in situ latrine sludge resistance. This investigation aimed to better understand the community education and technical engineering needs necessary to improve pit latrine management. In low income areas within Mzuzu city, Malawi, 300 pit latrines from three distinct areas were assessed using a dynamic cone penetrometer to quantify fecal sludge strength, and household members were surveyed to determine their knowledge of desludging procedures and practices likely to impact fecal sludge characteristics. The results demonstrate that there is a significant difference in sludge strength between lined and unlined pits within a defined area, though sludge hardened with depth, regardless of the pit type or region. There was only limited association between cone penetration depth and household survey data. To promote the adoption of pit emptying, it is recommended that households be provided with information that supports pit emptying, such as latrine construction designs, local pit emptying options, and cost. This study indicates that the use of a penetrometer test in the field prior to pit latrine emptying may facilitate the selection of appropriate pit emptying technology.

## 1. Introduction

Maintenance and management of on-site sanitation facilities (e.g., pit latrines) is a public health concern, as these facilities represent the main means of human waste collection for most of the population in the developing world [1]. The limited availability of land in peri-urban areas increases the need to find effective ways to empty existing pits rather than build new latrines. Pit emptying in the densely populated areas of Sub-Saharan Africa is a challenging task given the difficulty in accessing pit latrines, traffic congestion for haulage, and long travel distances to treatment plants. However, the health and environmental benefits of emptying pit latrines are significant [2]. Currently, there is limited knowledge of the physical properties of sludge in situ within pits, making the design and comparison of effective and durable pumping strategies difficult [3]. Additionally, the sludge contents of each pit latrine are different. For example, in South Africa, a study of sludge characterization described variability in moisture content, total and volatile solids, chemical oxygen demand, and aerobic biodegradability [4].

More recent efforts to characterize sludge combine techniques from different fields of research. Radford and Fenner [3] describe the use of geotechnical principles in fecal sludge characterization through the comparison of fecal sludge to weak soils using penetration resistance as a measure of shear strength. The Dynamic Cone Penetration Test (DCPT) is commonly used to determine engineering parameters, including resistance values with depth. Standard in situ resistance tests involve the repeated application of a replicable force in a probing process, which aims to characterize the resistance of the strata and identify potential obstacles by observing penetration resistance [5]. Currently, there is limited information on DCPT in many developing countries because its application is new in the sanitation field, though in theory resistance determined by a penetrometer should also be related to the pumpability or “fluidity” of fecal sludge.

Manual penetrometer procedures have been successfully used to measure sludge strength in Nairobi, Kenya and in Kampala, Uganda [6,7]. Both studies report great variability in sludge strength within the layers of pit latrines and provide operators engaged in sludge extraction with useful information on the challenges they may face, such as high strength sludge that may require fluidization before removal. Sludge density and shear strength are also dependent on organic content. For example, viscosity can increase over time due to decomposition [8], which may require operators to use various tools for sludge extraction.

The goals of this research are to (1) survey latrine construction and management in Mzuzu city, Malawi, with a focus on homeowner’s knowledge of desludging procedures and practices likely to impact fecal sludge characteristics and (2) quantitatively assess the resistance of fecal sludge in Mzuzu city, Malawi using a dynamic cone penetrometer procedure. The data obtained identify the community education and technical engineering needs necessary to improve pit latrine management. In addition, this work helps build the global knowledgebase on the variability of in situ latrine sludge characteristics.

## 2. Materials and Methods

Mzuzu city, located in northern Malawi, does not have a centralized wastewater treatment system or sewer. Therefore, households rely on either septic tanks or pit latrines for human waste disposal. Currently, there are no city or national regulations regarding the emptying of pit latrines and the City Council does not have a refuse collection and disposal system in place [9]. This study purposively targeted three different neighborhoods: (1) Luwinga, characterized by a relatively low water table (*n* = 100); (2) Ching’ambo, characterized by a high water table (*n* = 99); and (3) Masasa, characterized by steep slopes (*n* = 101). The Luwinga neighborhood is classified as a high density permanent area whereas Ching’ambo and Masasa are classified as informal/squatter areas [10]. The 300 households were selected without a preconceived conception of their accompanying latrine designs. Each survey consisted of a household questionnaire, field observations of the latrine design and its maintenance, and a penetrometer test of the pit latrine’s sludge resistance. The data were collected during the rainy season.

Participants were informed of their right to choose whether or not to take part in the research, and written informed consent was obtained via study protocols approved by the Republic of Malawi, National Commission for Science and Technology (Protocol No: P.12/15/69).

### 2.1. Assessment of Household Sanitation Practices

Prior to conducting each penetration test, a semi-structured household questionnaire was administered orally in the local language (Chichewa or Chitumbuka) to gather household demographics, sanitation practices, and an understanding of pit emptying. Questions were yes/no or multiple choice with additional open-ended follow-up questions to determine the reasons behind specific answers. The questionnaire also included a field observation checklist of the household’s pit latrine structure.

The questionnaire assessed general sanitation practices including solid waste management, the duration over which the latrine has been in use (based on recall), the occurrence of diarrhea in the last week (based on recall), and the materials used for anal cleansing after latrine use. An inspection of the latrine design collected information on the location of the latrine, the characteristics of the latrine slab, and the general characteristics of the latrine housing.

### 2.2. Assessment of Sludge Strength

**Figure 1:**
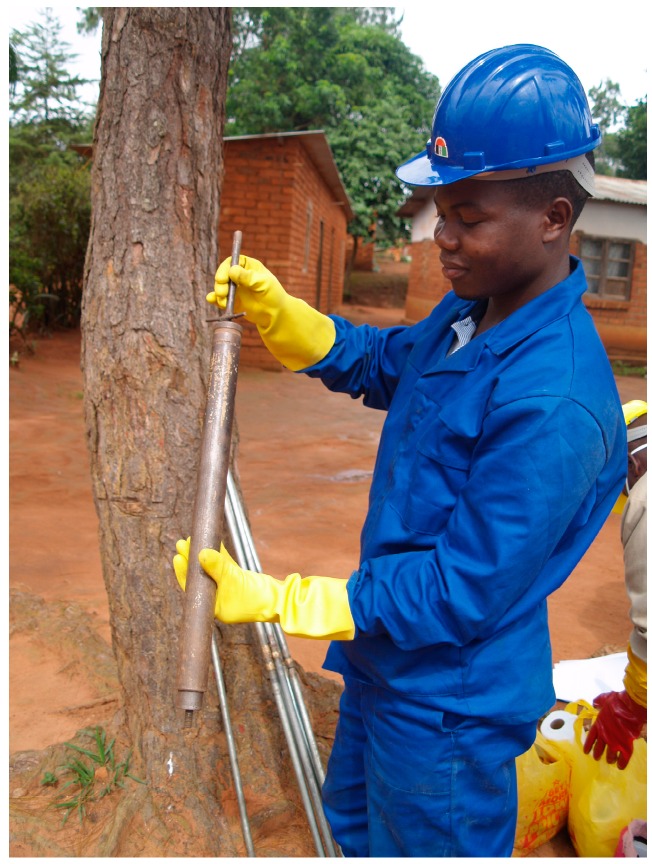
Dynamic cone penetrometer.

The manual cone penetrometer (North Carolina State University, Raleigh, NC, USA) test was completed either on the day of the household survey, or the day after (Figure 1). Field procedures for in situ resistance determination of fecal sludge were based on methodologies previously used in Nairobi, Kenya [7] and Kampala, Uganda [6]. The testing method for the use of a dynamic cone penetrometer (designation D6951/D6951 M-09 [11]) was generally used to ensure that the in situ resistance could be calculated based on the penetration resistance of the materials, though our penetrometer was a different size. This study used a penetration cone 55 mm long and 35 mm in diameter at the base of a 15 mm diameter rod (that had up to six 600 mm long sections with an average weight of 0.33 kg each and total depth maximum of 3600 mm) with an adjustable penetration depth based on the number of rods connected. At the beginning of testing in each latrine, the cone penetrometer joined to the cone rod was lowered slowly until it touched the sludge under the slab in order to measure the depth of the sludge below the pit latrine floor. After this, the cone penetrometer was left to settle on its own without applying force. Once it stopped, a reference point was marked on the rods remaining above the slab. A consistent impact was applied using a sliding impact hammer with a weight of 1.9 kg dropped from a predetermined height of 540 mm onto an anvil which weighed 0.35 kg. The reference point was used to measure the penetration depth after the application of a consistent impact against a reference point marked on the latrine slab. The test involved driving the cone penetrometer into the undisturbed fecal sludge within the pit using repeated blows from a falling hammer. In cases where penetration was beyond the first reference point on the rod, more rods were added and a new reference point was marked on the added rod.

To establish the correct number of impacts, two pit latrines were pretested before beginning the initial tests. The overall penetration results are based on the average of three penetrometer tests in each pit latrine, located at the front, middle, and back of the pit latrine squat (key) hole. The pit latrine squat (key) hole is centrally positioned inside the latrine. From the pre-test results, each penetrometer test consisted of three sets of five-impacts each followed by a set of 10-impacts until full penetration was achieved. The application of the impacts was stopped if no further penetration was achieved after applying a combination of three sets of the 10-impact sequence (30 blows). After penetrometer testing, the cone penetration rod was cleaned using a bleach solution (sodium hypochlorite) in the retrieval process.

**Figure 2:**
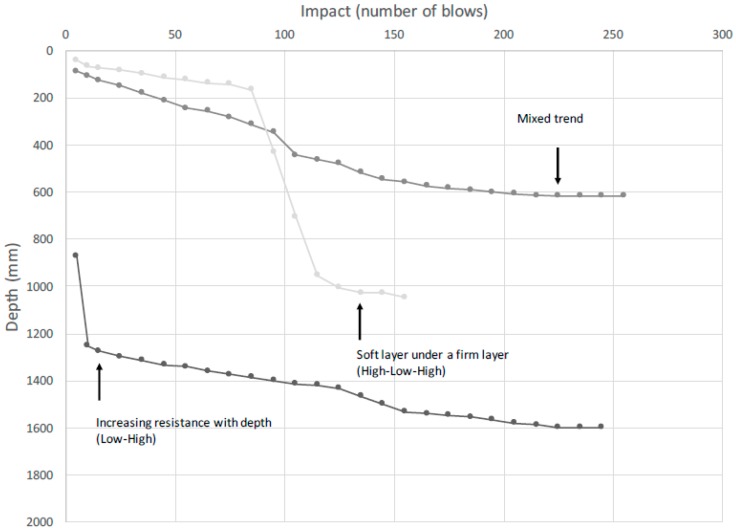
Representative sludge strength with depth classification based on method from Seal [7].

To analyze penetrometer results, the penetration rate was calculated at every depth for each test (as deep as the test went), then averaged to obtain the penetration rate by depth. The results are presented as mm/blow. Trends in penetration resistance with increasing depth were categorized and based on the method used by Seal [7] (Figure 2).

## 3. Results

### 3.1. Household Sanitation Practices

Household sanitation practices varied by neighborhood, as evidenced by the survey results (Table 1). Respondents indicated their method for anal cleansing included toilet paper/tissues or water, maize cobs, newspaper, and cardboard. The majority of respondents used materials other than paper/tissues or water for anal cleansing, a practice likely to impact pit latrine characteristics. Questioning the household’s perception of pit emptying practices using open ended queries revealed that pit emptying was perceived as a process meant for septic tanks only, a perception that has prevented respondents from calling for emptying services. Approximately 85% (256/300) of the households surveyed responded that they would likely dig another latrine when their pit was full. This finding is important for city fecal sludge managers and planners so that they can understand the sanitation issues that need to be addressed in order to increase the demand for pit emptying services in low income areas.

Unlined pit latrines present the risk that the pit latrine walls may collapse during regular use or emptying. Observations of latrine conditions revealed that only 5% (15/300) of the latrine substructure (below ground surface pit) walls were plastered with cement. Around one half (57%; 172/300) of the tested latrines had permanent superstructures (walls for privacy) with walls made of local bricks and 46% (138/300) had a cement floor or slab. Cracks were observed in 11% (34/300) of the surveyed latrine floors. The presence of cracks may indicate that the pit latrine floor is not strong enough to hold the weight of the pit latrine emptying operator and/or equipment. Squat (key) hole covers and handwashing facilities (within 5 m) were found in 21% (64/300) and 21% (64/300), respectively.

For households indicating knowledge of pit latrine sludge emptying (79%; 237/300), the source of information was mainly a neighbor/friend/family member (61%; 144/237), followed by personal observation (21%; 50/237) and the media (11%; 25/237). The majority of studied households also perceived pit emptying as an expensive procedure and only 29% (86/300) expressed their willingness to pay for emptying services, but typically did not have information on the costs of pit emptying services and the available options.

Households using a latrine that was almost full (i.e., the latrine had sludge within 100 mm of the latrine floor) showed a prevalence in Luwinga of 7% (7/100), in Ching’ambo of 20% (20/99), and Masasa of 1% (1/101). The higher percentage in Ching’ambo can be explained by floating pit waste due to the high water table and the study timing during the rainy season.

### 3.2. Sludge Strength Results

#### 3.2.1. Impact-Based Analysis

The impact depth procedure measures the depth achieved by the cone penetrometer after a number of impacts from the hammer. At a typical pit latrine, the penetration testing took 13 min. Table 2 presents the results for penetration levels achieved by specific impact counts. The results show variability in sludge strength within and between areas. Five hammer blows achieved a maximum average penetration depth in Luwinga of 533 mm for unlined pits and 647 mm for lined, in Ching’ambo of 420 mm for unlined and 1163 mm for lined, and in Masasa of 587 mm for unlined and 113 mm for lined.

Overall, there are differences in penetration depth between lined and unlined pit latrines. The results from Ching’ambo revealed a rapid decrease in penetration depth after the first five impacts. In addition, unlined pits in the Ching’ambo area had a smaller range between the depth reached by 5 and 35 impacts, which relates to the existing high water table conditions that contribute to low sludge strength near the surface. This also has an influence on the maximum depth reached by the first five impacts, which is considerably higher in Ching’ambo lined latrines compared to the Luwinga and Masasa areas.

#### 3.2.2. Penetration Resistance

Penetration depth was affected by the presence of trash, as visually observed during the field penetration procedures. Trash ranged from pieces of cloth to cans that interfered with the penetration measurements during the in situ tests. Although only a few respondents indicated household waste was put into the pit latrine, the field observations were to the contrary. This finding is also combined with most respondents indicating anal cleansing with a range of materials other than toilet paper/tissue or water.

**Figure 3:**
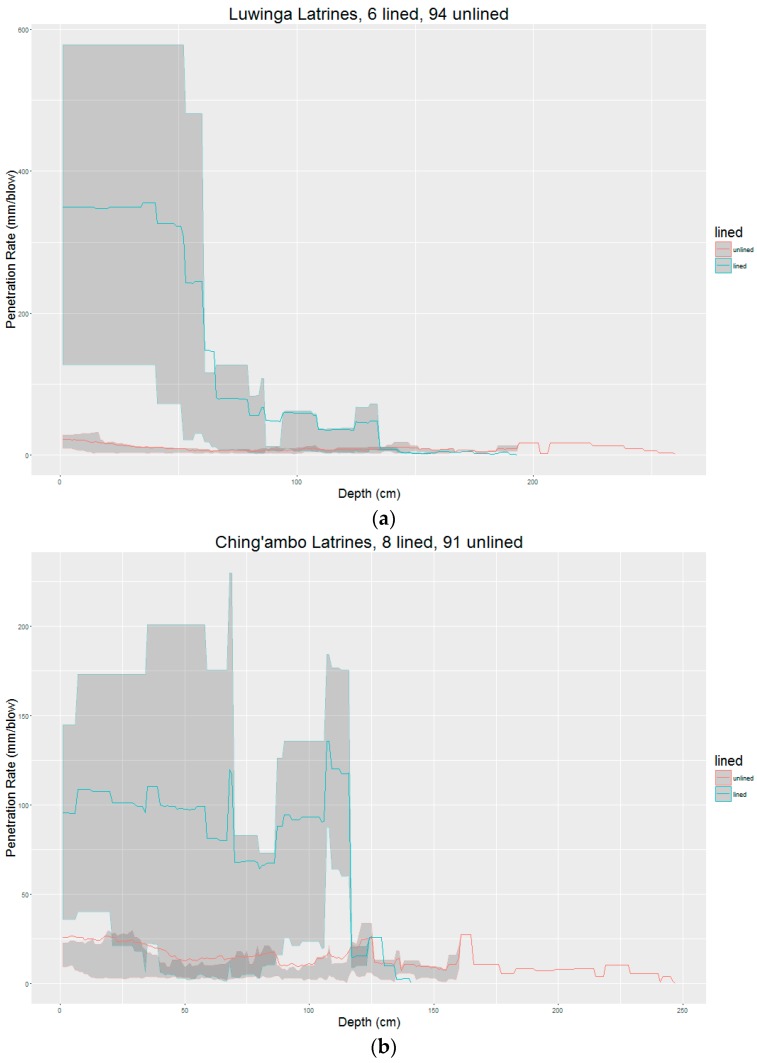

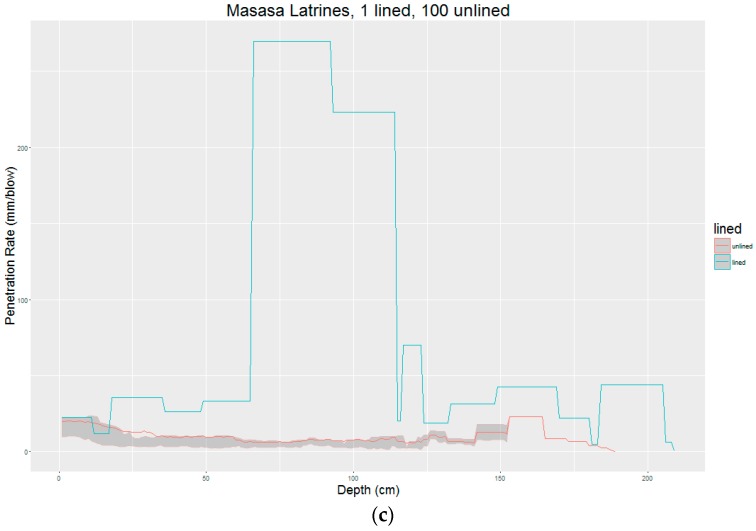
Average sludge strength for lined latrines and unlined latrines in areas of (**a**) Luwinga; (**b**) Ching’ambo; and (**c**) Masasa. Shading indicates 75th and 25th percentiles.

Average sludge resistance of lined and unlined latrines are compared across the three locations in Figure 3. Solid lines indicate the mean penetration rate (mm/blow) at a given depth, whereas shading denotes the 75th and 25th percentiles, providing an indication of the range of penetration rate values observed. The study results indicate that the resistance of sludge in the sampled latrines increases on average with increasing depth. Furthermore, as shown in Figure 3, and as tested statistically at 25 cm and 50 cm, sludge resistance is much higher for unlined latrines than for lined latrines. For unlined pit latrines, sludge strength variation was generally limited within a region, within the range of 20 to 50 mm per blow. However, this observation did not occur in all latrines. In some unlined latrines, the sludge near the surface was soft and the strength increased rapidly with depth. While in others, some low and high resistance sludge occurred throughout the pit depth. Sludge became harder with depth, regardless of the pit type or region. The regression lines have negative slopes showing an inverse relationship between sludge strength and mm/blow. In other words, the deeper the penetrometer went, the fewer mm/blow were possible through the sludge. Therefore, there is a negative association between mm/blow and depth, but a positive association between sludge strength and depth.

In Luwinga, 6/100 latrines were lined. Based on the overall trend of penetration resistance, unlined latrines in the Luwinga area had relatively lower resistance near the surface, which generally increased with depth. The best lines of fit for lined and unlined pits had slopes of −2.734 and −0.092, respectively, so that for a 1 cm increase in depth, the penetration rate decreased by 2.734 mm/blow and 0.092 mm/blow for lined and unlined pits, respectively. These results indicate an increase in sludge strength with an increase in depth. Comparing the penetration rate between lined and unlined pits for Luwinga at a depth of 25 cm showed that there is a significant difference in penetration resistance (*p*-value = 0.014).

In Ching’ambo (*n* = 99), 8% of the latrines were lined and 92% were unlined. As illustrated by Figure 3, the penetration resistance graphs for lined and unlined pit latrines in Ching’ambo followed similar sludge resistance trends; in both cases, the sludge near the surface was soft and the resistance increased with the increasing depth. Statistical results showed a positive association between sludge strength and depth. Similarly to Luwinga, the linear regression best lines of fit had negative slopes, −0.394 and −0.121 for lined and unlined pits, respectively. Additionally, there was a significant difference in penetration resistance at a depth of 25 cm between lined and unlined sludge (*p*-value = 0.039).

The Masasa area had only one lined pit latrine. Masasa area also had a high number of reported pit latrine collapses from respondents. Unlike the Luwinga and Ching’ambo areas, the soft surface in unlined pits in Masasa was observed within a depth of less than 0.5 m, which indicates a rapid increase in sludge strength. Overall, the association between sludge strength and depth was positive, similar to the Luwinga and Ching’ambo areas, with linear regression slopes of −0.187 and −0.122 for lined and unlined latrines, respectively. This may relate to a high drainage rate due to the steep terrain in the area resulting in high sludge strength.

**Figure 4:**
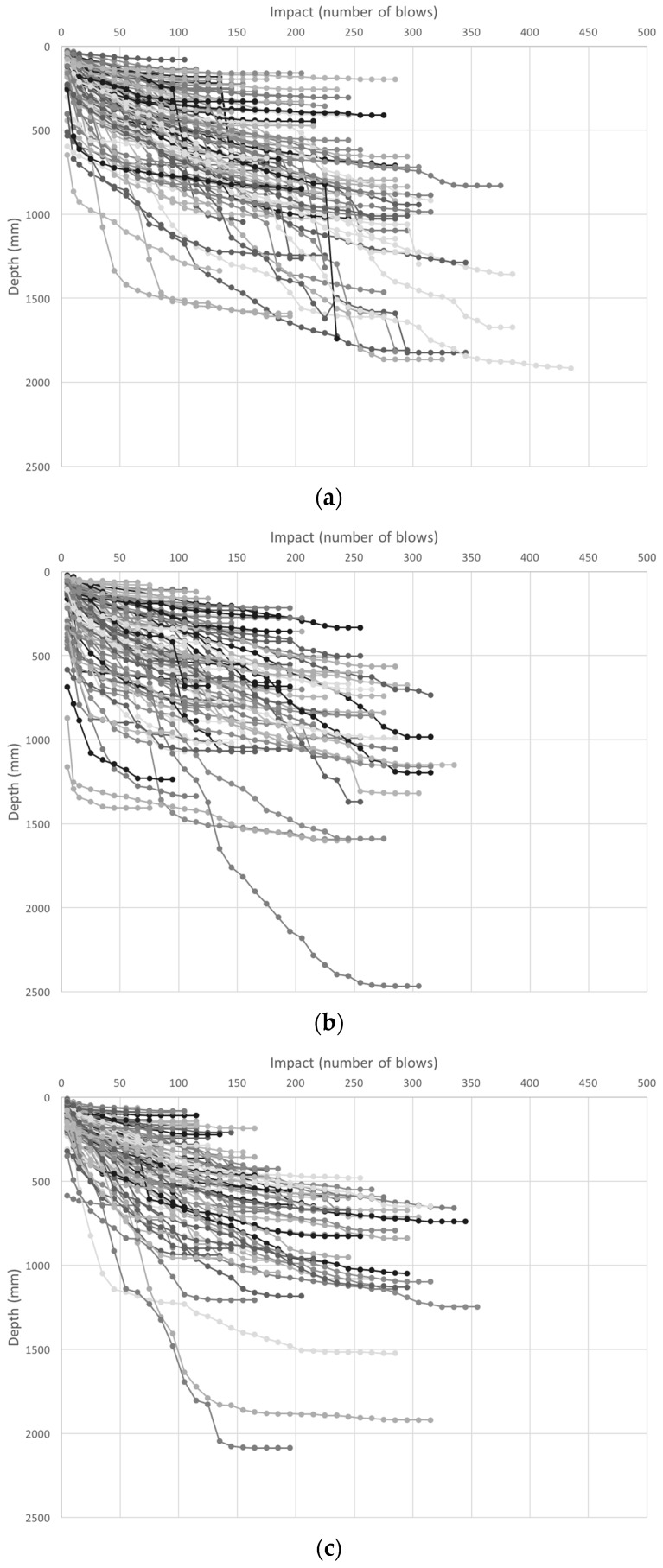
Sludge strength variation with depth in areas of (**a**) Luwinga; (**b**) Ching’ambo; and (**c**) Masasa. Lines represent individual test results in each area.

Soft sludge layers are characterized by a sharp increase of penetrometer depth verses impact, whereas hard layers are characterized by a flatter slope (Figure 4). The low to high resistance trend was most common in both Ching’ambo (observed in 57/99 latrines) and Masasa (observed in 59/101 latrines). A comparison of the three areas showed that low resistance sludge existed over a larger depth in Ching’ambo compared to the other two areas. The mixed trend with depth was most common in Luwinga (observed in 49/100 latrines). A high-low-high resistance trend, characterized by a firm top layer followed by a soft middle layer then a firm bottom layer, was the least common trend in each of the three areas. Low resistance sludge (a soft layer) on top of the sludge may be easily removed using manual or mechanical pit emptying tools, whereas high resistance sludge (high strength) layers that account for the majority of pit latrine volume may require the use of specialized pit-emptying techniques such as fluidization prior to emptying operations.

The only survey data related to the sludge penetration rate data was that at a depth of 100 cm (*n* = 57), latrines of households reporting they used only toilet paper had a 37 mm/blow higher penetration rate than latrines of households reporting they used any source of paper including newspaper. No other variables (besides lined/unlined) were statistically significant indicators of penetration rate (sludge strength).

The study results indicate that the resistance of sludge in the sampled latrines is dependent on the pit type and groundwater conditions.

#### 3.2.3. Depth Effect Analysis

Despite the interference from trash, based on the classification method in Figure 2, most of the latrines tended to be soft on top and the strength increased with depth, as shown in Figure 4. This is supported by regression analysis, as indicated by the negative slope in the best fit line. In Ching’ambo, the soft layer stretched over a greater depth compared to measurements from Luwinga and Masasa. In general, the slope of the soft layer was consistent between the three trends. However, after the soft layer, different sludge characteristics were found with increasing latrine depth. Some latrines had a profile where the strength increased steadily with depth, while in other latrines the sludge exhibited well-defined layers that occurred at different levels in different orders. The latrines with the low-to-high trend had the deepest penetration profiles.

## 4. Discussion

Overall, this study suggests that fecal sludge resistance is greatly influenced by pit type and existing groundwater conditions, but there was only limited association with household survey data. This study observed that most people were not willing to pay for emptying services. However, the area of Ching’ambo, characterized by a high water table, indicated a higher demand at 57% (56/99), and 84% (83/99) of households had knowledge of pit emptying. This area also had the highest number of lined latrines of the three areas. This is a positive sign for pit emptying businesses. A household’s willingness to empty their existing pit may also relate to the difficulty of digging a new pit latrine in an area with a high water table. However, marketing strategies are needed to help address user concerns such as emptying costs from service providers. A high water table may have an effect on the filling rate of the pit latrine [12]. Households in high density areas with a high water table in Kampala, Uganda have been found to use less expensive manual emptiers that usually dump fecal sludge in an open environment [13], though a comparison in Mzuzu of the operation of manual emptiers requires further research.

The Ching’ambo area had the highest proportion of latrine superstructures which were mainly made of plastic bags and pieces of timber, and had clay floors. The high proportion of latrines without a cement floor slab means that these latrines may not be able to support the weight of some emptying equipment such as the modified gulper [14].

Making pit emptying service information available to households, especially in high water table areas, may help to increase the adoption of pit emptying and management in Mzuzu. As indicated by the survey results, most households (61%) received pit emptying information from informal sources such as friends, which may mean the information is incomplete or biased. This lack of information may also contribute to the low utilization of emptying services, as households do not have complete information regarding the benefits of pit emptying. Our results also correlate to results in Tanzania, where it has also been reported that households delay emptying and continue to use full pits beyond what is safe [15].

As explained by Strande et al. [1], pit wall collapse is a sign of the low bearing capacity of the soils surrounding the latrine substructure walls, a physical hazard that interferes with pit emptying. Respondent reports of collapsed pit latrines may create a barrier to latrine use and force households to select the perceived lower risk practice of open defecation, consistent with findings from Obeng et al. [16]. This problem was observed within our study especially in the Masasa area. High numbers of pit collapses may be linked to households unlikely to pay for emptying services, and such conditions additionally make emptying of pit latrines dangerous.

As observed in Figure 3, the thickness of low resistance sludge layers in unlined pits was different between the three study areas. The low resistance sludge (near the surface) in Ching’ambo occurred over a larger depth compared to the sludge in Luwinga and Masasa where the low resistance sludge occurred in narrower layers. The difference in the thickness of the soft sludge layer could be attributed to the difference in groundwater movement and the impact of consolidation as the sludge was undisturbed [17]. The low resistance sludge in Ching’ambo is consistent with Radford and Fenner [3] who explain the dilution effect of water on fecal sludge, and by Buckley et al. [18] who observed the reduction in sludge resistance resulting from an increase in water content caused by net movement of water into the pits located in high water table areas. Our results indicate a single tool for pit emptying in Mzuzu may be inadequate due to the variability of fecal sludge resistance.

The findings of this study are also consistent with results from Seal [7], which refutes that pit latrines are soft at the surface with gradually increasing density towards the bottom. In this study, observations were not uniform across all latrines, since some latrines had low resistance sludge at different depths below the surface. The dissimilarity in sludge resistance between the top and deep sludge layers can be explained by the influence of compaction and aging processes on sludge characteristics, which result in the formation of different sludge layers that exhibit different characteristics. However, this observation cannot be attributed to compaction effects alone because strength varies with latrine depth and layers of different characteristics were observed at different levels. The penetration is also affected by the obstacles throughout the penetration depth, including solid waste and historical pit collapse. As data were collected during the rainy season, further research is suggested for penetrometer testing during the dry season to account for shallow groundwater variations.

Observations of lined latrines were similar in all areas; however, it is worth noting that there were no more than 10 lined pit latrines in any of the study areas. The high resistance (hard) sludge in this study found in unlined pits may only be removed by vacuum based emptying technologies [3,8]. Additionally, in places such as Mzuzu, where not all areas are accessible to vacuum trucks, removal of thick sludge with trash at the bottom of pits may require manual emptying or fluidization, thus increasing the operational cost for emptying [3]. Fluidization during pit emptying also requires additional equipment, expertise, and labor. Related findings were also observed by Semiyaga et al. [2] who discussed the impact of increased waste production in urban areas and poor urban waste management on fecal sludge characteristics. Non-degradable materials in pit latrines also affect the secondary treatment of the sludge, resulting in increased sludge management costs [2,15].

The manual cone penetrometer was a relatively easy to use field tool to test the resistance of in situ fecal sludge. Our larger diameter cone at 35 mm, compared to other dynamic cone penetrometers [11], is better for the weak materials in a pit latrine. However, a study limitation is that the zone of influence around the cone is limited. In situ samples are needed to determine the constants used in shear strength calculations. With standardization, the manual cone penetrometer could be an important field tool for developing real time knowledge of physical properties of fecal sludge in other low-income countries.

## 5. Conclusions

Great variability in pit latrine sludge strength was observed in three areas of the city of Mzuzu. Low sludge resistance layers mainly occurred near the surface while lower depths exhibited relatively higher resistance (dense) sludge. The testing revealed a high portion of latrines that contained trash, which is a sign of poor household waste management. The sludge characteristics of lined and unlined pits are different and therefore likely to have different emptying technical needs; the value of using the penetrometer is that emptying service providers can tease out these differences and target them appropriately.

Considering there is no program to phase out pit latrines in Malawi, it is recommended that future fecal sludge management and planning strategies for pit latrine emptying in Mzuzu be combined with the promotion of lined household pit latrine designs, education on household waste management (such as in combination with Urban Community Led Total Sanitation campaigns), plus marketing of local pit latrine emptying options and costs. Additionally, the penetrometer data should be combined with analytical results of sludge solid content (during emptying) and indicators of trash content with depth. This study indicates that the use of the dynamic cone penetrometer test in the field prior to pit latrine emptying may assist with the selection of appropriate pit emptying technology.

## Figures and Tables

**Table 1:** Household sanitation practices and knowledge of pit latrine emptying (*n* = 300).

Characteristic	Response Categories	Luwinga (*n* = 100)	Ching’ambo (*n* = 99)	Masasa (*n* = 101)
Religious affiliation	Christianity	100%	98%	97%
	Muslim	-	2%	3%
Household incidence of diarrhea within the past seven days	No	84%	89%	93%
	Yes	16%	11%	7%
Household waste management practices	In the pit latrine	1%	-	-
	In water	-	1%	3%
	Rubbish pit	95%	81%	70%
	Surface	4%	18%	27%
Time over which the latrine has been in use	>2 Years	65%	81%	71%
	1–2 Years	30%	19%	29%
	<1 year	-	-	-
	Unknown	5%	-	
Method used for anal cleansing	Toilet paper/tissue only	14%	11%	9%
	Any materials other than toilet paper/tissue or water	86%	87%	90%
	Water	-	2%	1%
Sharing of the latrine facility with other households	Not shared	49%	35%	40%
	Shared	51%	65%	60%
Household option in case of a full pit latrine	Call emptying service	15%	17%	3%
	Dig another pit	80%	80%	96%
	Use chemicals	2%	2%	-
	Use neighbor’s latrine	3%	1%	1%
Household knowledge of pit latrine sludge emptying	No knowledge	35%	16%	12%
	Knowledgeable	65%	84%	88%
Willingness to pay for emptying services		24%	57%	6%

**Table 2:** Sludge strength based on impact count.

Area	Number of Impacts	Mean Depth (mm)		Maximum Depth (mm)		Minimum Depth (mm)	
		U	L	U	L	U	L
Luwinga (*n* = 100)	5	100	451	533	647	20	143
	35	283	824	697	1077	57	563
	75	408	1057	1268	1480	77	793
Ching’ambo (*n* = 99)	5	101	554	420	1163	20	20
	35	297	916	1057	1400	57	420
	75	424	1055	1287	1407	80	730
Masasa * (*n* = 101)	5	93	113	587		10	
	35	271	647	1050		60	
	75	416	1230	1206		80	

U = Unlined; L = Lined; * In Masasa there was only one lined pit latrine.
